# Supplementary material for: Strengthening phage resistance of Streptococcus thermophilus by leveraging complementary defense systems
Source: Nat Commun. 2025 Aug 4;16:7142. doi: 10.1038/s41467-025-62408-3 (PMC12322248; doi:10.1038/s41467-025-62408-3)
Supplement: Supplementary file 1 — Supplementary information [file 41467_2025_62408_MOESM1_ESM.pdf]

Strengthening Phage Resistance of *Streptococcus thermophilus* by Leveraging Complementary Defense Systems

Audrey Leprince, Justine Lefrançois, Anne M. Millen, Damian Magill, Philippe Horvath, Dennis A. Romero, Geneviève M. Rousseau and Sylvain Moineau.

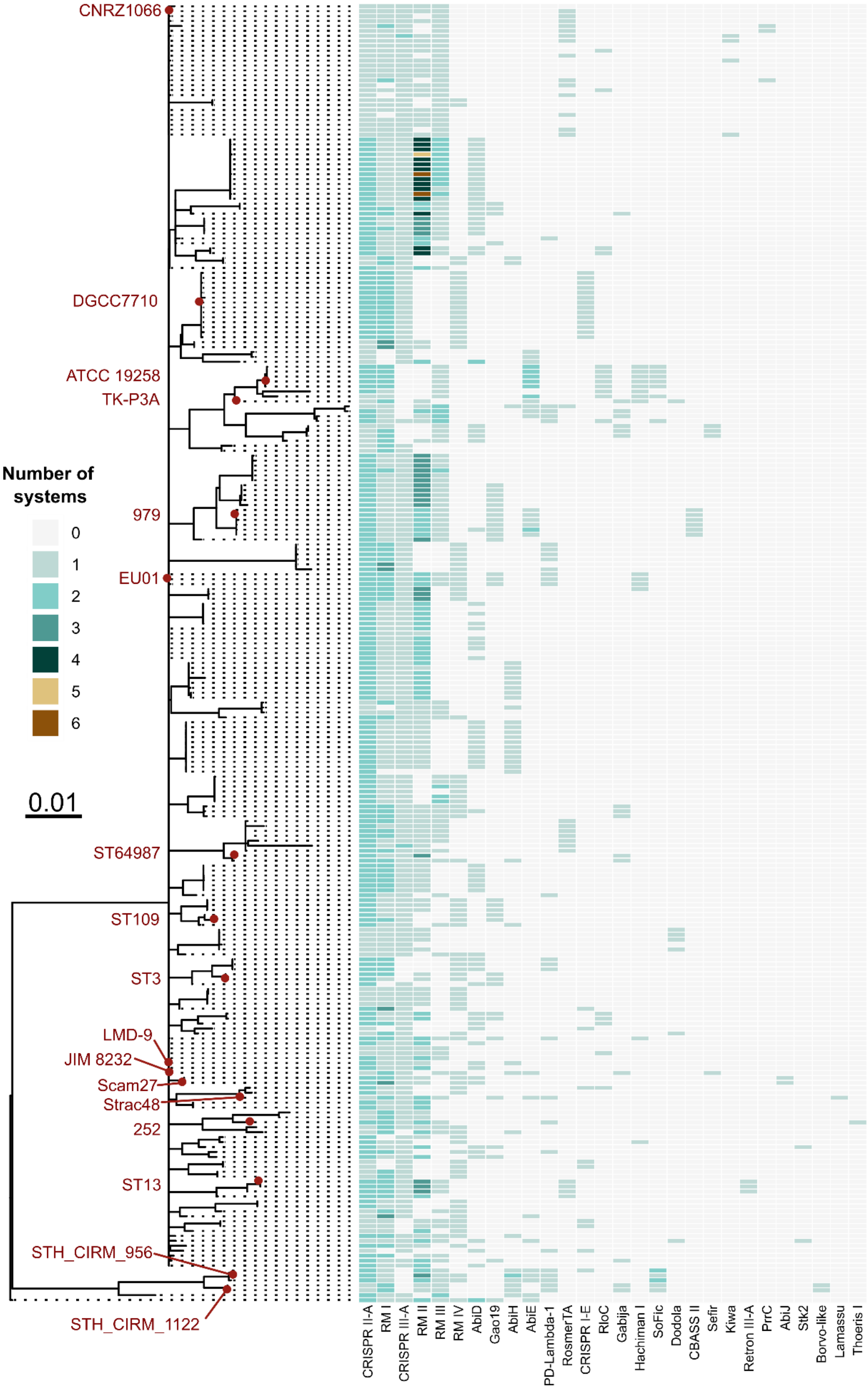

Supplementary Figure 1 - Phylogenetic tree of *S. thermophilus* genomes and their defense systems. The heatmap indicates the presence and number of defense systems within each genome. Strains with defense systems that were used in the experimental part of this study are highlighted in red. For additional details, refer to Tables S3 and S7. Branch lengths represent the number of amino acid substitutions per site. Source data are provided as a Source Data file.

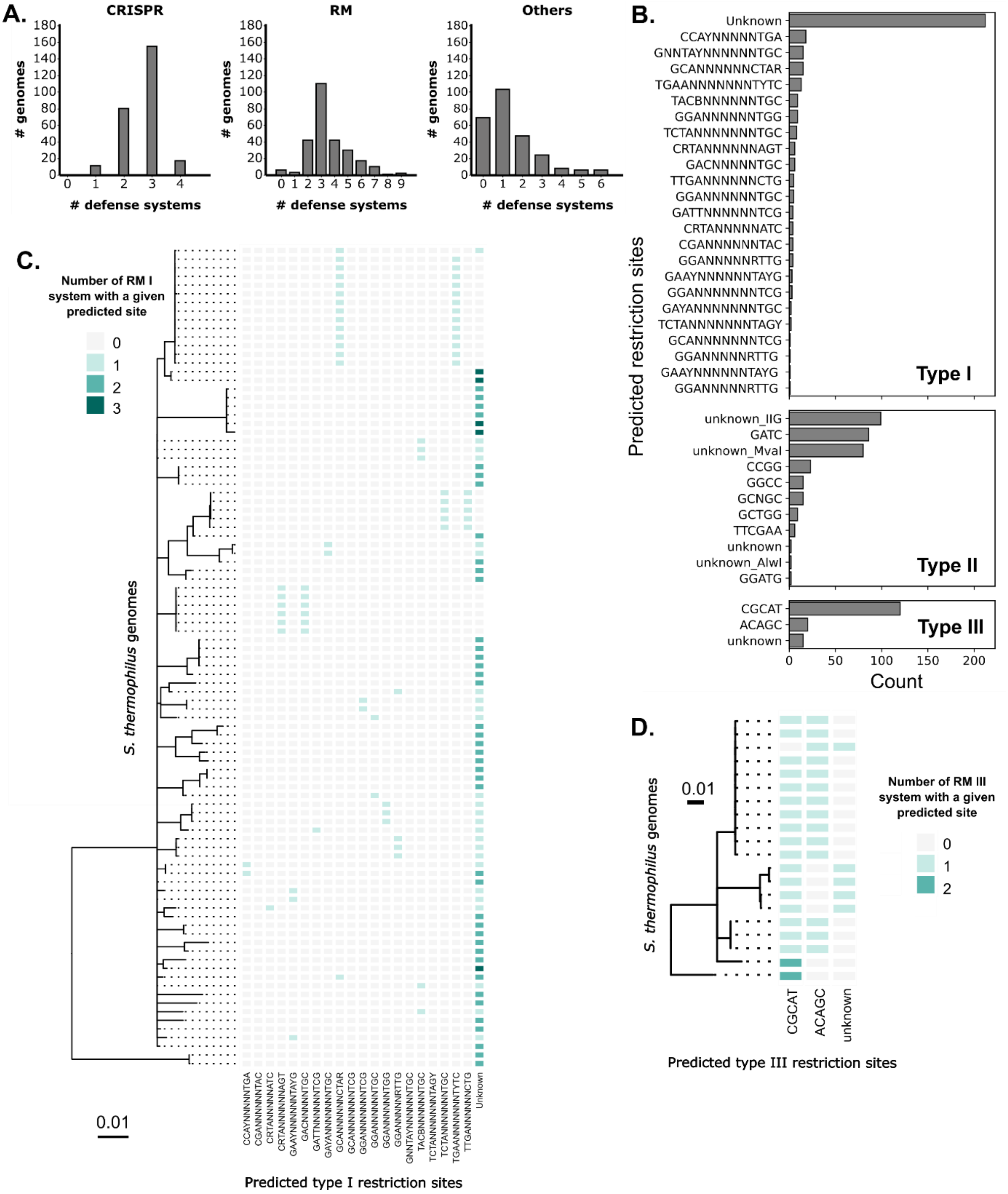

Supplementary Figure 2 - Additional information on the prevalence of defense systems and RM-predicted recognition sites. A. Histograms showing the number of defense systems per strain according to the categories CRISPR, RM, and other systems. **B.** Prevalence of each predicted restriction site according to the RM type. For type II systems, the category “unknown” was divided into “unknown_MvaII”, “unknown_AlwI”, “unknown_IIG”, and “unknown” based on the restriction enzyme annotation (MvaI/BcnI family or AlwI family) or RM type II (type IIG). **C-D.** Heatmaps displaying co-occurrence within the same genome of RM type I (**C.**) and type III systems (**D.**) with different predicted restriction sites. Only *S. thermophilus* strains with more than two RM type I or RM type III systems are shown and represented on the phylogenetic tree. Branch lengths represent the number of amino acid substitutions per site. RM: Restriction-modification. Source data are provided as a Source Data file.

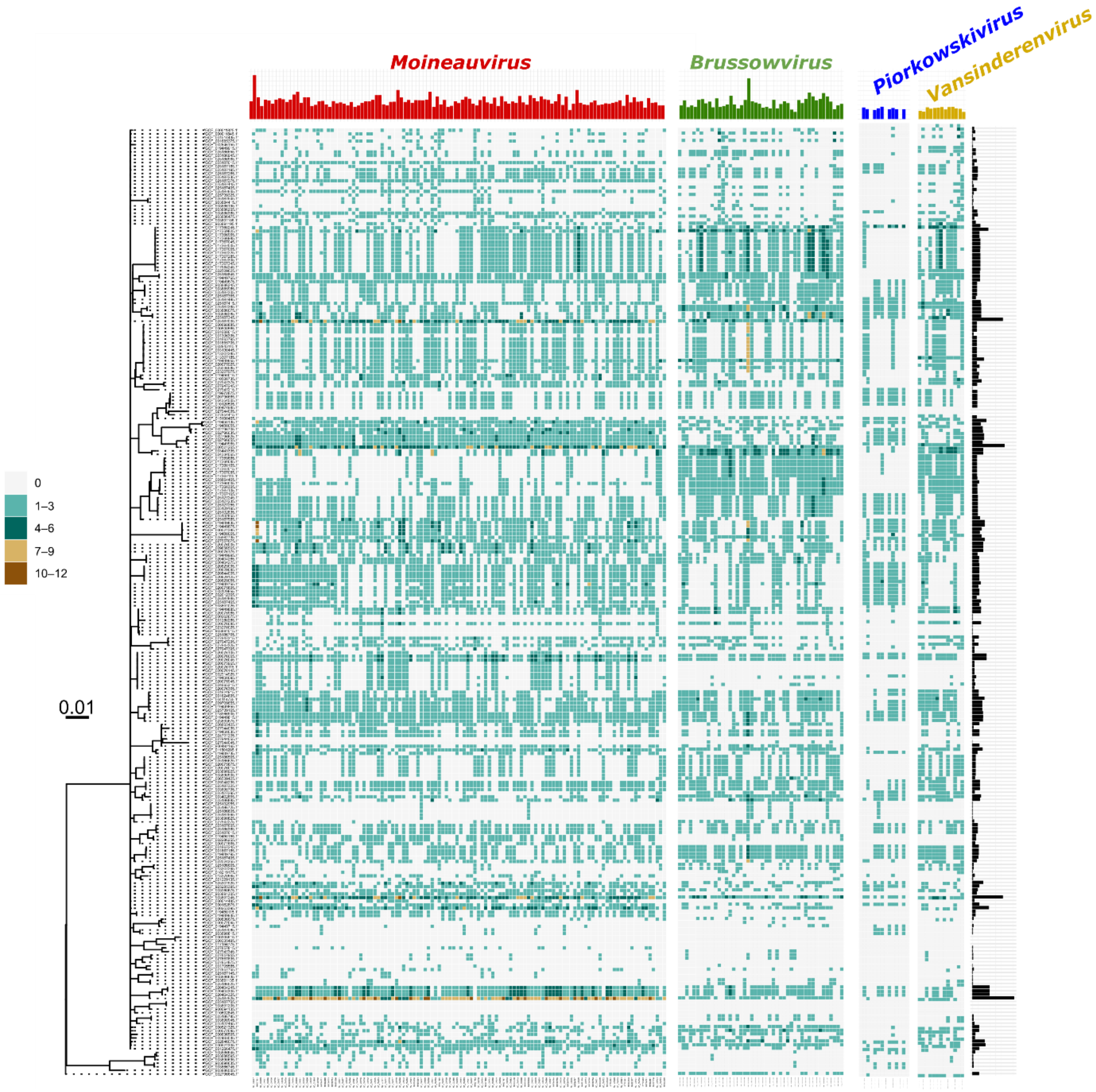

Supplementary Figure 3 - Analysis of spacer–protospacer dynamics in the CR1 loci of *S. thermophilus* strains. Spacers were extracted from the CR1 loci of the 263 *S. thermophilus* strains and compared to a custom database of 191 phage genomes using BLASTn. The phylogenetic tree on the left illustrates the relationships between the bacterial strains, with branch lengths representing the number of amino acid substitutions per site. Phages are shown along the x-axis. The heatmap displays the number of spacers per strain with a 100% match to protospacers in the corresponding phages, as indicated by the color scale. The bar plot above the heatmap indicates the total number of matching spacers per phage (range: 0–450), while the bar plot to the right shows the total number of matching spacers per strain (range: 0–880). Source data are provided as a Source Data file.

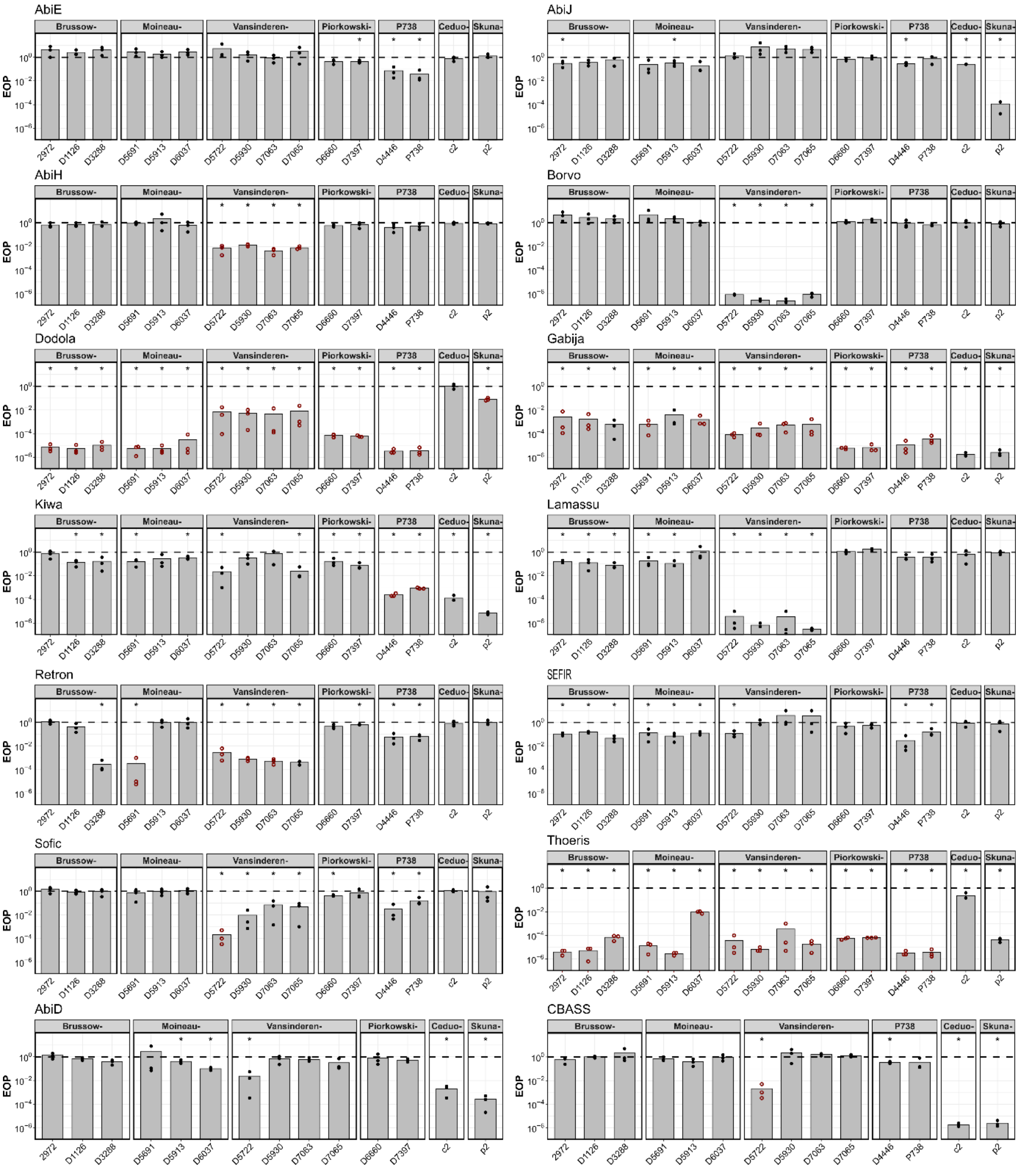

Supplementary Figure 4 - Detailed spot test results using streptococcal and lactococcal phages for defense systems for which a single homologue was tested. Bars represent the mean EOP ± SD (error bars) from biological replicates (n=3) which are each represented by a circle. Filled circles indicate the presence of countable plaques, while hollow red circles signify zones of lysis where plaques were not observed. The dotted line represents an EOP of 1, indicating no phage defense. Asterisks indicate statistical significance (EOP different from 1) according to a one-sample, two-sided t-test with a p-value less than 0.05 and multiple testing corrections (Benjamini-Hochberg) were applied. Hyphen on top of each graph replaces the word *virus*. EOP: Efficiency of plaquing. Source data are provided as a Source Data file.

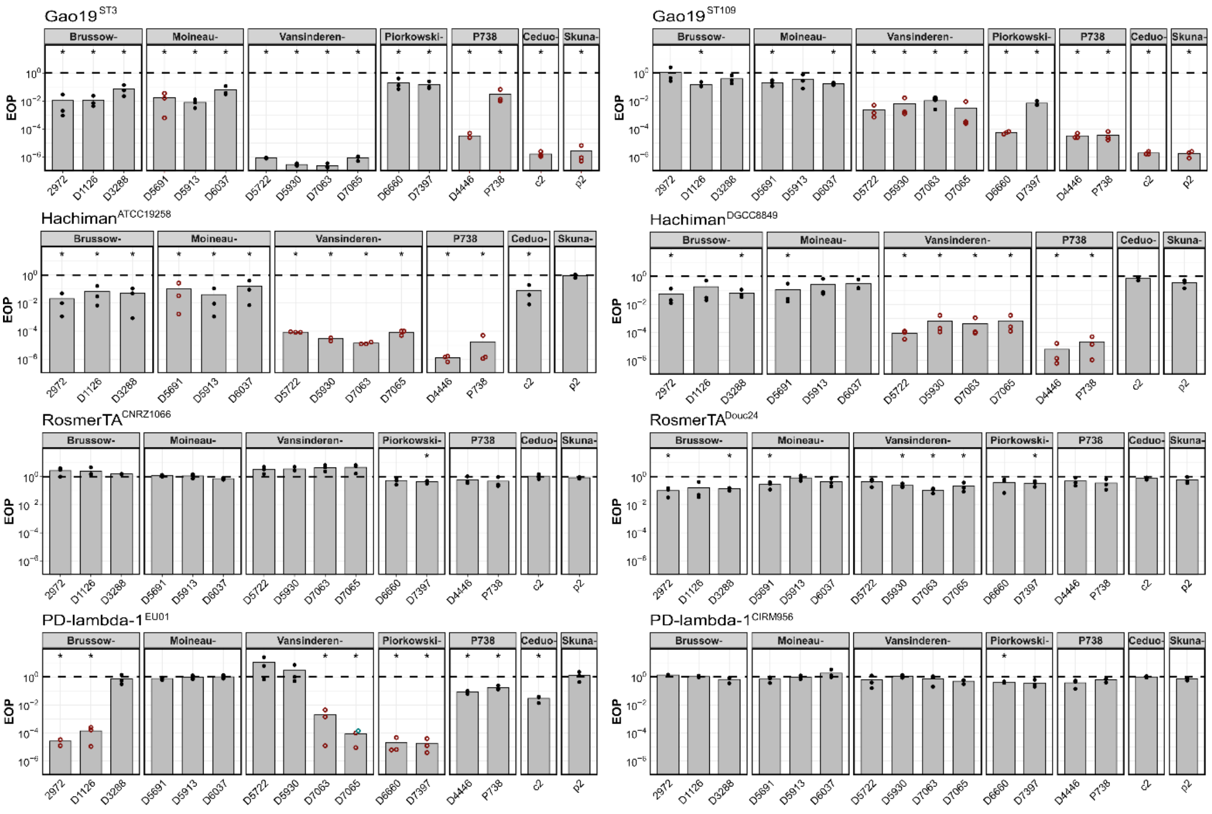

Supplementary Figure 5 - Detailed spot test results using streptococcal and lactococcal phages for defense systems for which two homologues were tested. Bars represent the mean EOP ± SD (error bars) from biological replicates (n=3) which are each represented by a circle. Filled circles indicate the presence of countable plaques, while hollow red circles signify zones of lysis where plaques were not observed. The dotted line represents an EOP of 1, indicating no phage defense. Asterisks indicate statistical significance (EOP different from 1) according to a one-sample, two-sided t-test with a p-value less than 0.05 and multiple testing corrections (Benjamini-Hochberg) were applied. Hyphen on top of each graph replaces the word *virus*. EOP: Efficiency of plaquing. Source data are provided as a Source Data file.

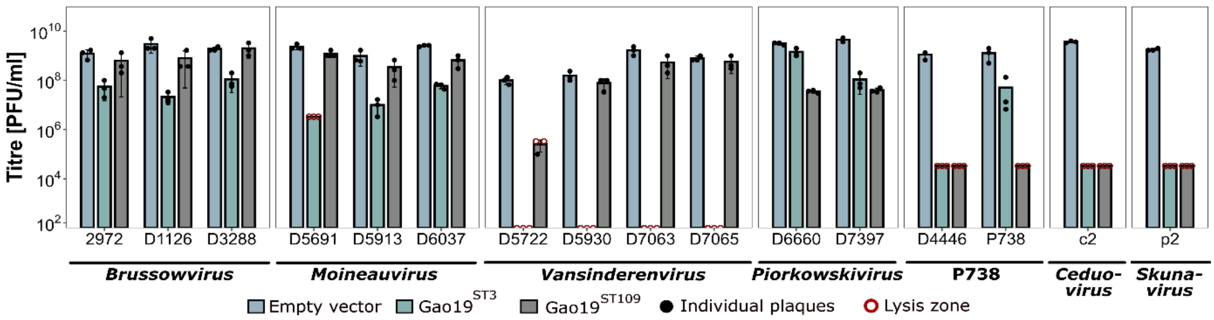

Supplementary Figure 6 – Graphs showing the efficiency of Gao19 homologs infecting *S. thermophilus*, measured as plaque-forming units (PFU) per mL. The homologs from *S. thermophilus* strains ST3 and ST09 were tested against the five genera infecting *S. thermophilus* and the two *L. cremoris* phages (c2 and p2). Bars represent the mean titre ± SD (error bars) from biological replicates (n=3) which are each represented by a circle. Filled circles indicate the presence of countable plaques, while hollow red circles signify zones of lysis where plaques were not observed. Source data are provided as a Source Data file.

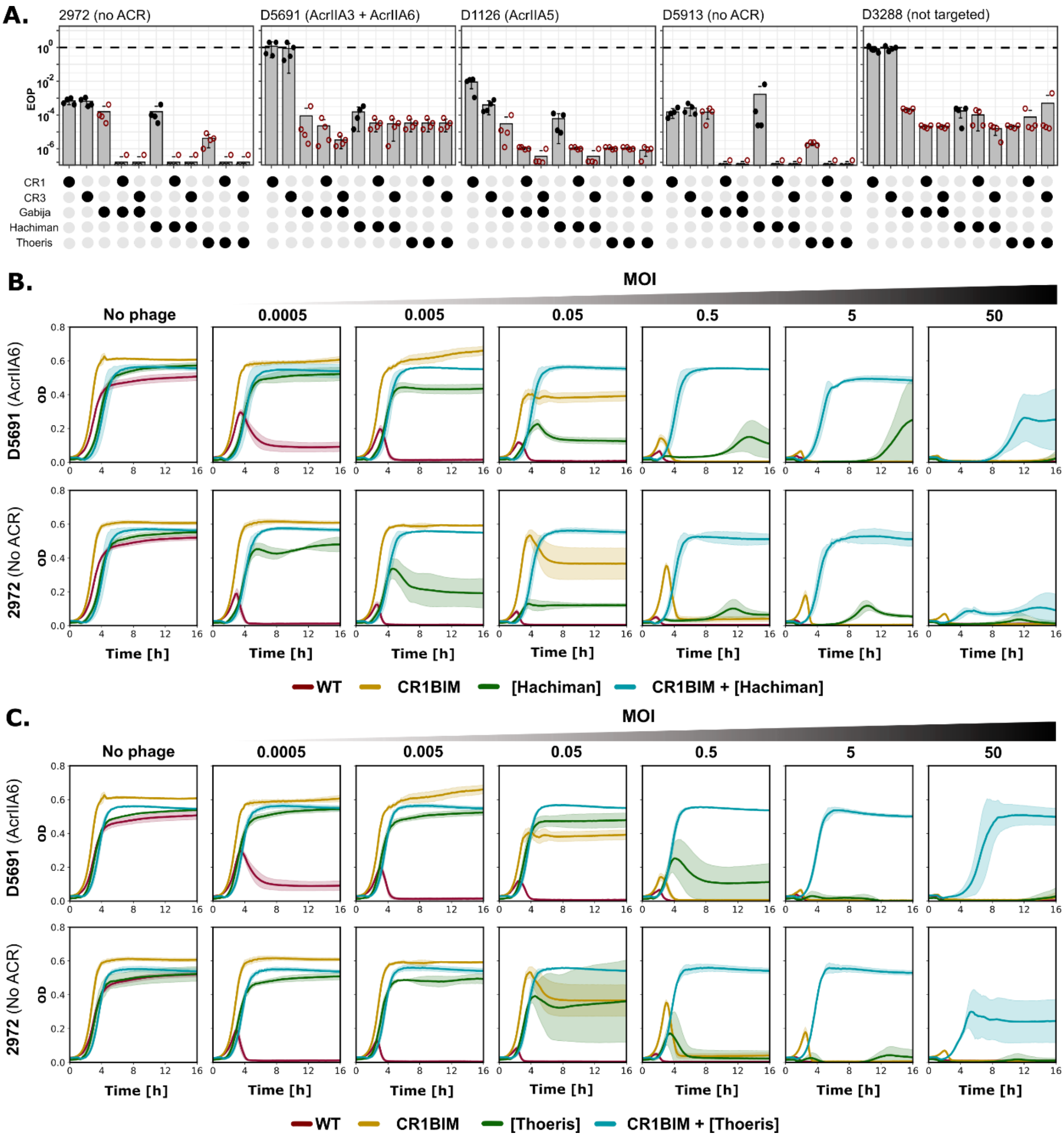

Supplementary Figure 7 - Phage resistance conferred by combining CRISPR-Cas systems with additional defense mechanisms. A. Detailed spot test results for combinations of Gabija, Hachiman, or Thoeris with either CR1 or CR3 immunity. Five phages were tested: D5691 which encodes an AcrIIA3 (anti-CR3 activity) and AcRIIA6 (anti-CR1 activity), D1126 which encodes an AcrIIA5 (anti-CR1 and anti-CR3 activities), 2972 and D5913 which do not carry known ACRs, and D3288 which is not targeted by the spacers in the CR1- and CR3-immune strains. Bars represent the mean EOP from biological replicates (n=3), which are each represented by a circle and error bars indicate standard deviations. Filled circles indicate the presence of countable plaques, while hollow red circles signify zones of lysis where plaques were not observed. The dotted line represents an EOP of 1, indicating no phage resistance. **B-C.** Killing assays comparing CR1 + Hachiman (**B**) or CR1 + Thoeris (**C**) combinations against phages 2972 and D5691 at MOIs ranging from 0.0005 to 50. Control strains include wild-type DGCC7710 (WT) and CR1-immune DGCC7710 (CR1BIM) carrying the empty pTRKL2 vector. The defense system provided *in trans* via the pTRKL2 plasmid is indicated in square brackets. Each line represents the mean of independent replicates (n=3); shaded areas indicate confidence intervals. ACR: Anti-CRISPR protein; OD: Optical density; EOP: Efficiency of Plaquing. Source data are provided as a Source Data file.

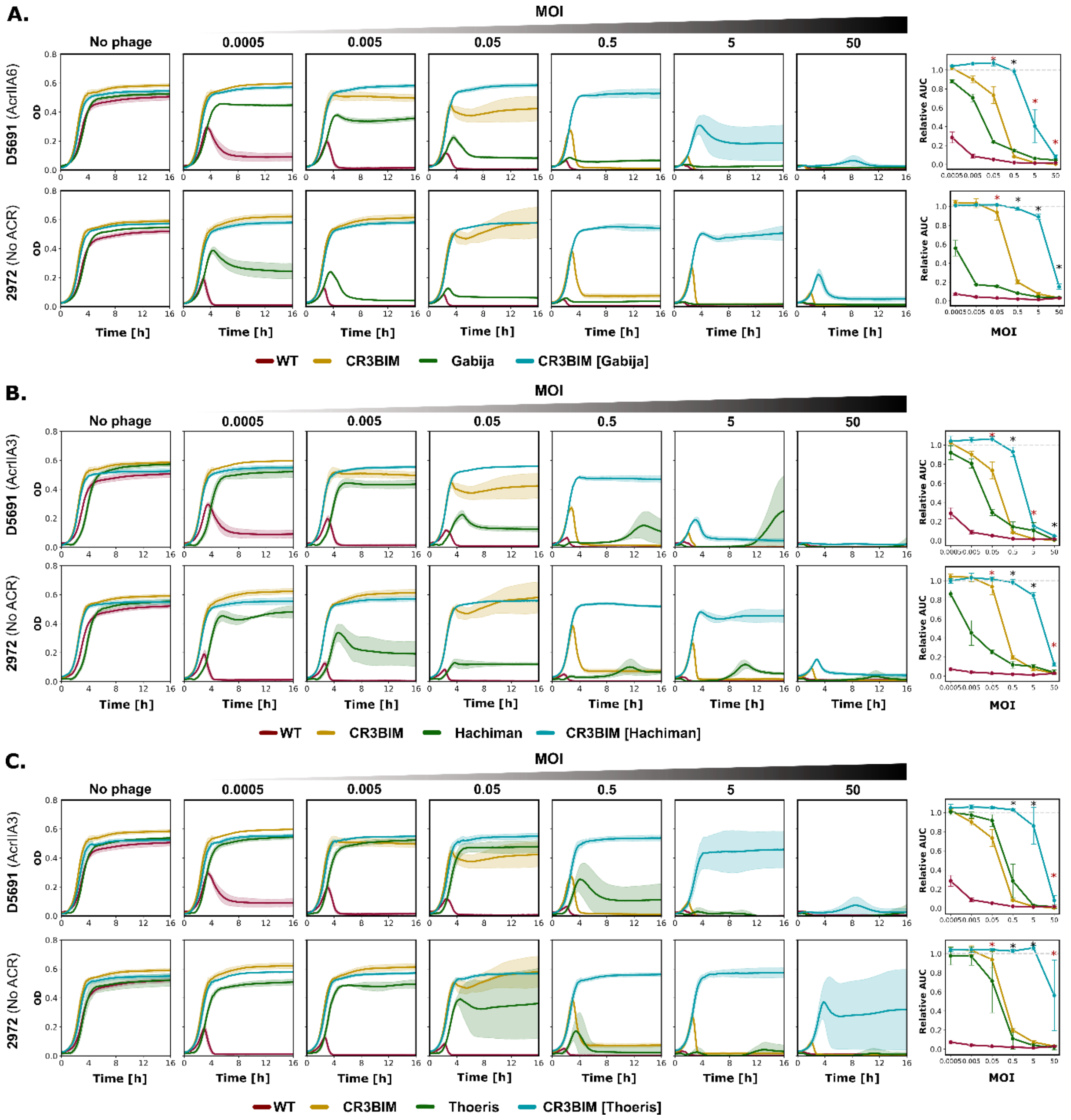

Supplementary Figure 8 - Liquid assay results for CR3 immunity combined with additional defense systems. Killing assays were performed to evaluate the combined effect of CR3 immunity with either Gabija (**A**), Hachiman (**B**), or Thoeris (**C**) against phages 2972 and D5691, across a range of MOIs (0.0005 to 50). Phage 2972 lacks known anti-CRISPR proteins, while phage D5691 encodes AcrIIA6 (inhibits CR1) and AcrIIA3 (inhibits CR3). The area under the curve (AUC) was calculated for each condition and plotted against MOI. Control strains include wild-type phage-sensitive strain DGCC7710 (WT) and CR3-immune DGCC7710 (CR3BIM), both carrying the empty pTRKL2 vector. The defense system provided *in trans* via the pTRKL2 plasmid is indicated in square brackets. For all experiments, lines or bars represent the mean value of biological replicates (n=3), with shaded areas indicating confidence intervals and error bars indicating standard deviation. The statistical significance of the synergy scores was evaluated using a two-sided one-sample t-test with Benjamini-Hochberg correction for multiple testing. A positive score significantly different from zero (p-value < 0.05) indicated synergy (black asterisk), while a score not significantly different from zero indicated an additive effect (red asterisk). ACR: Anti-CRISPR protein; MOI: Multiplicity of Infection; OD: Optical density. Source data are provided as a Source Data file.

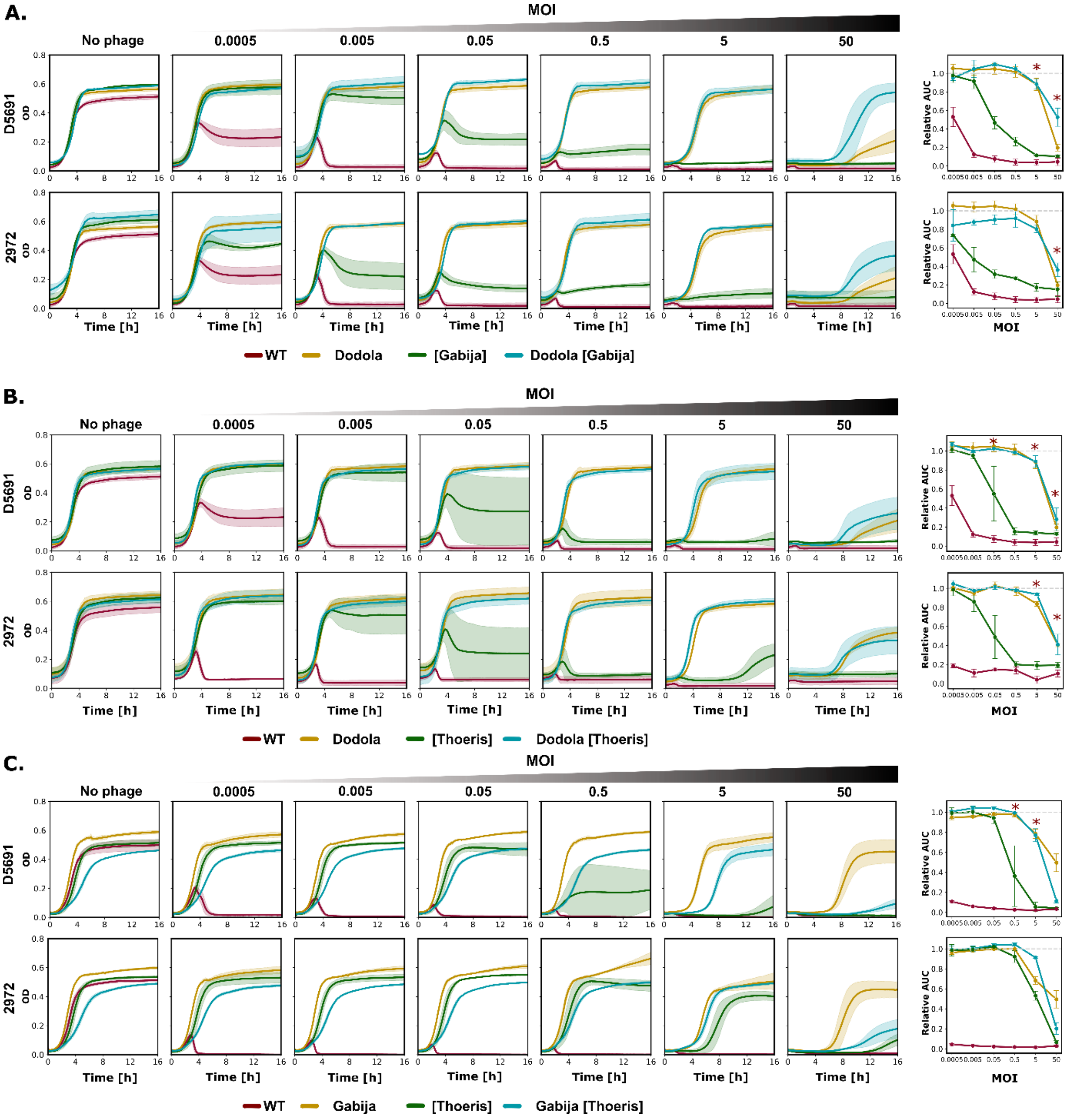

Supplementary Figure 9 - Liquid culture assays evaluating combinations of non-CRISPR defense systems. Killing assays were performed to evaluate the combined effect of different pairs of defense system, namely Dodola-Gabija (**A**), Dodola-Thoeris (**B**), and Gabija-Thoeris (**C**), against phages 2972 and D5691, across a range of MOIs (0.0005 to 50). In each combination, one defense system (Dodola or Gabija) was chromosomally integrated, while the second was expressed in trans from the pTRKL2 vector (indicated in square brackets). The area under the curve (AUC) was calculated for each condition and plotted against MOI. Control strains include wild-type phage-sensitive DGCC7710 (WT) carrying the empty pTRKL2 vector. For all experiments, lines or bars represent the mean value of biological replicates (n=3), with shaded areas indicating confidence intervals and error bars indicating standard deviation. The statistical significance of the synergy scores was evaluated using a two-sided one-sample t-test with Benjamini-Hochberg correction for multiple testing. A positive score significantly different from zero (p-value < 0.05) indicated synergy (black asterisk), while a score not significantly different from zero indicated an additive effect (red asterisk). MOI: Multiplicity of Infection; OD: Optical density. Source data are provided as a Source Data file.

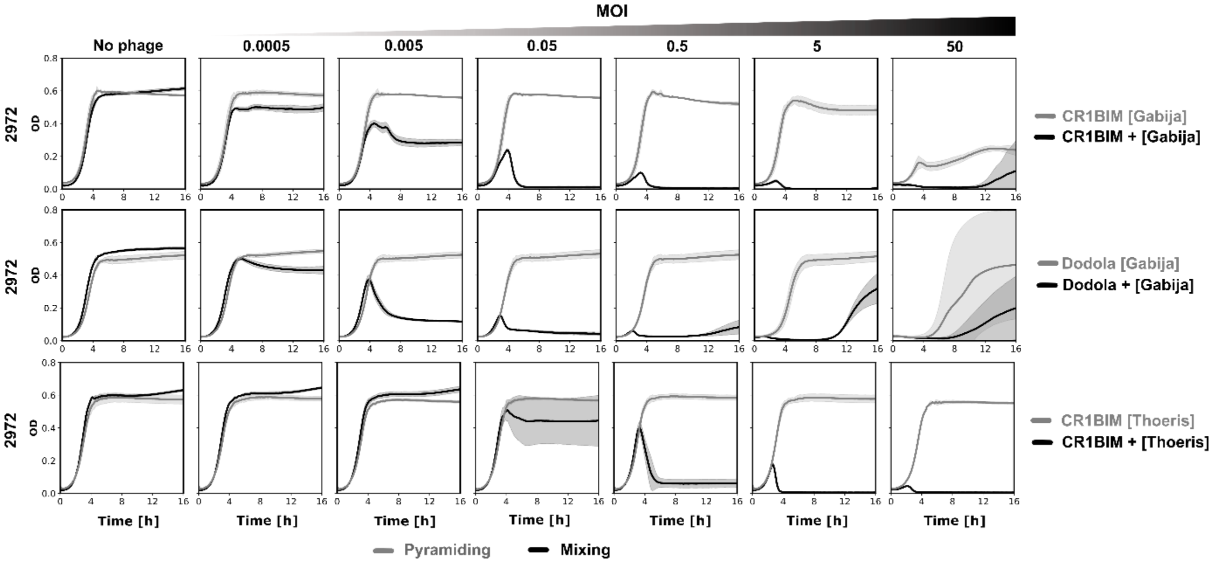

Supplementary Figure 10 - Killing assay results comparing pyramiding and mixing strategies using phage 2972 across a range of MOIs (0.0005 to 50). This figure presents detailed results from liquid culture assays designed to evaluate the effectiveness of *pyramiding* versus *mixing* strategies for combining defense systems. In the *pyramiding* approach, all cells in the population carry both defense systems of interest. In contrast, the *mixing* strategy involves a 1:1 mixture of two strains, each carrying only one of the individual defense systems. Three defense system pairs were tested, namely CR1 + Gabija, Dodola + Gabija, and CR1 + Thoeris. The defense system provided *in trans* via the pTRKL2 plasmid is indicated in square brackets. Each curve represents the mean of biological replicates (n=3) and shaded areas show confidence intervals. OD: Optical density. Source data are provided as a Source Data file.

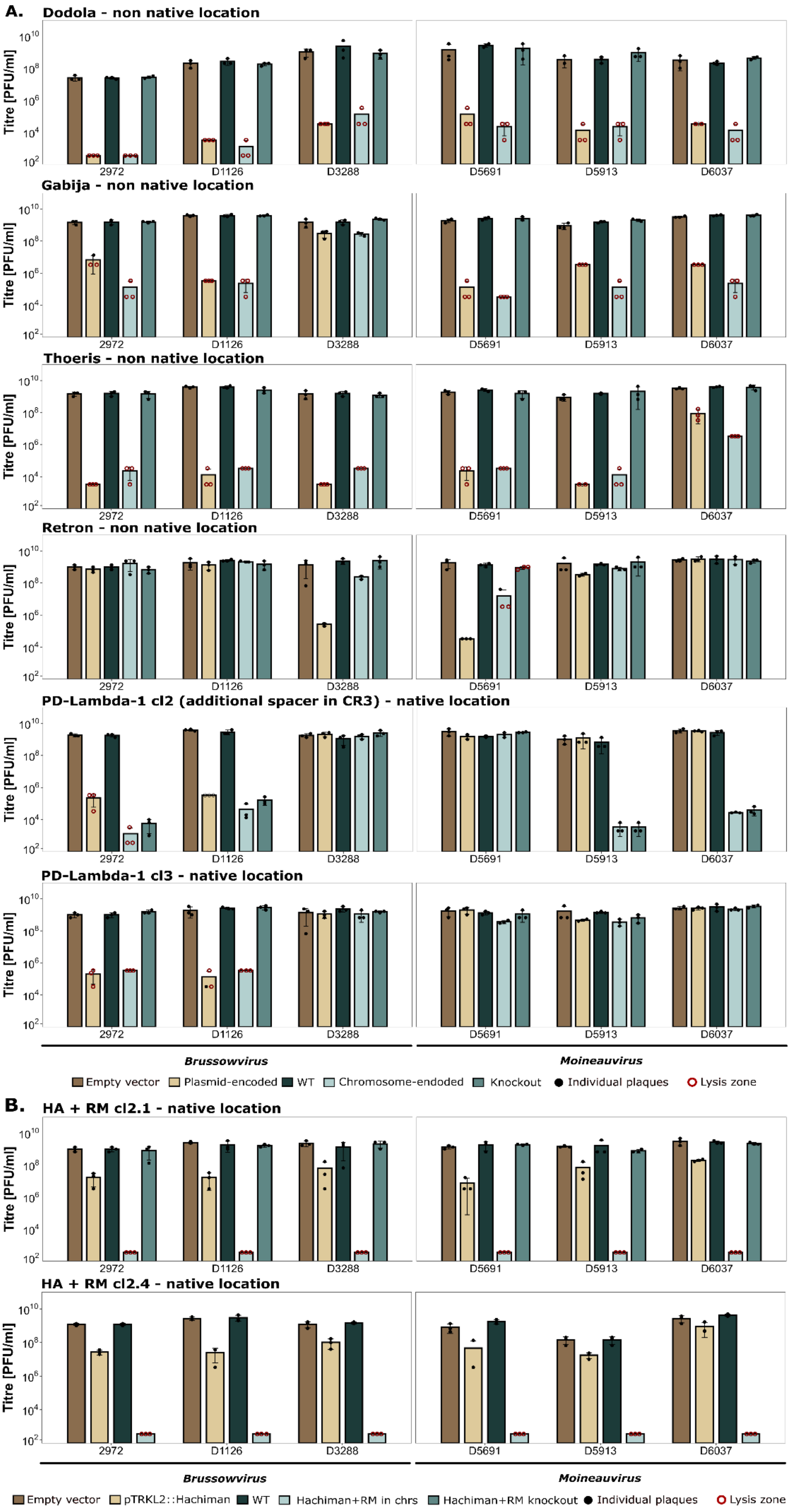

A.
Dodola - non native location
Gabija - non native location
Thoeris - non native location
Retron - non native location
PD-Lambda-1 cl2 (additional spacer in CR3) - native location
PD-Lambda-1 cl3 - native location
Titre [PFU/ml]
2972
D1126
D3288
D5691
D5913
D6037
Brussowvirus
Moineauvirus
Empty vector
Plasmid-encoded
WT
Chromosome-endoded
Knockout
Individual plaques
Lysis zone
B.
HA + RM cl2.1 - native location
HA + RM cl2.4 - native location
Empty vector
pTRKL2::Hachiman
WT
Hachiman+RM in chrs
Hachiman+RM knockout
Individual plaques
Lysis zone

Supplementary Figure S11 – Titers graphs showing the efficiency of defense systems when chromosomally integrated vs plasmid-encoded. A. Comparison between chromosomally integrated defense systems in *S. thermophilus* DGCC7710 and their plasmid-encoded counterparts against phages belonging to the *Brussowvirus* and *Moineauvirus* phage genera. The integration into a non native or native chromosomal location is indicated next to the defense system name. **B.** Analysis of the efficiency of a chromosomally integrated defense island (native location) containing Hachiman and a type II RM system in DGCC7710 against *Brussowvirus* and *Moineauvirus*. The efficiency of Hachiman alone when expressed from a pTRKL2 plasmid is also shown. For all experiments, bars represent the mean titre ± SD (error bars) from biological replicates (n=3) which are each represented by a circle. Filled circles indicate the presence of countable plaques, while hollow red circles signify zones of lysis where plaques were not observed. WT: Wild type; chrs: chromosome- encoded; cl: clone. Source data are provided as a Source Data file.

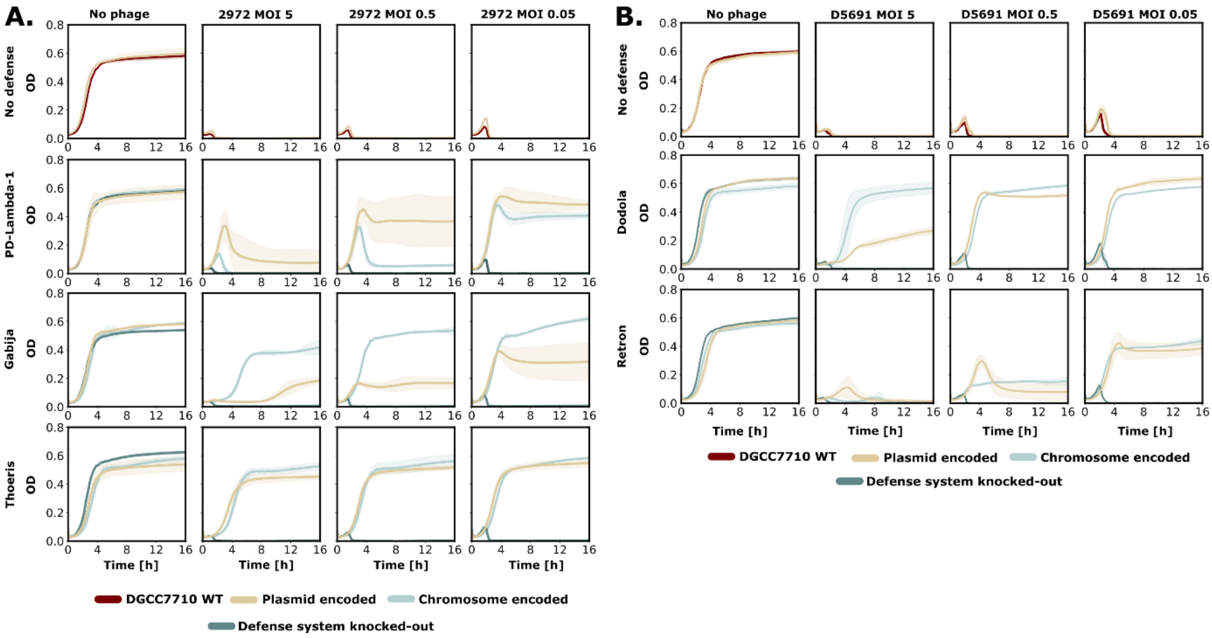

Supplementary Figure 12 - Comparison of the efficiency of chromosomally encoded versus plasmid-encoded defense systems against phages in liquid assays. Defense systems were tested in LM17 medium at 37 °C over 16 hours using three multiplicities of infection (MOIs: 0.05, 0.5, and 5) against either phage 2972 (**A**) or phage D5691 (**B**). Chromosomally integrated defense systems were also knocked out to assess the loss of phage protection. Each curve represent the mean of biological replicates (n=2) with shading area indicating confidence interval. OD: Optical density; MOI: Multiplicity of Infection; WT: wild-type. Source data are provided as a Source Data file.

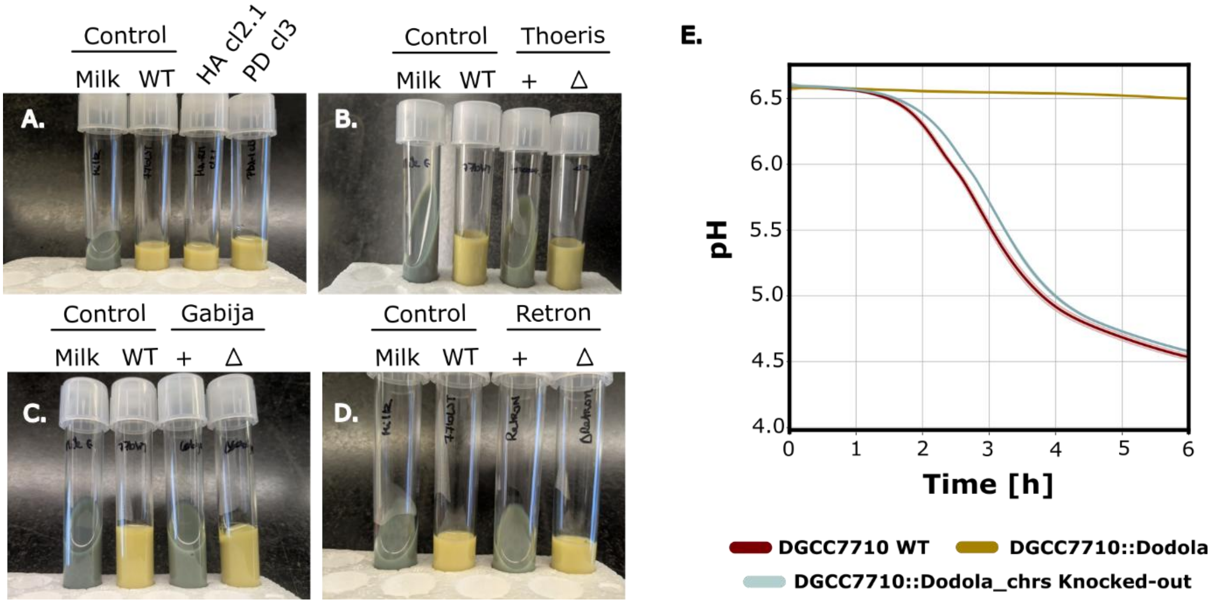

Supplementary Figure 13 - Milk acidification assays with *S. thermophilus* DGCC7710 containing chromosomally encoded defense systems. A. Milk coagulation assays with *S. thermophilus* DGCC7710 strains carrying the PD-Lambda-1 defense system (PD cl3) or a defense island composed of Hachiman and a type II RM system (HA cl2.1) integrated at their native chromosomal loci. Control conditions included "Milk" (uninoculated milk supplemented with 0.001% bromocresol purple) and "WT" (milk inoculated with the wild-type strain *S. thermophilus* DGCC7710). In the absence of milk fermentation, the milk remains blue/purple and liquid (Milk control), whereas fermentation leads to lactic acid production, lower pH, a yellow color, and milk coagulation (WT control). **B–D.** Milk coagulation assays with derivatives of *S. thermophilus* DGCC7710) containing an integrated defense systems at non-native chromosomal loci: Thoeris (**B**), Gabija (**C**), and Retron (**D**). **E.** pH monitoring over 6 hours of milk inoculated with the wild-type strain DGCC7710, a derivative DGCC7710 with chromosomally integrated Dodola, and its corresponding knockout derivative. Technical replicates (n=3) were done but one of DGCC7710::Dodola replicate was excluded due to an issue with the pH probe. Curves represent mean values with shaded arear showing the confidence interval. WT: wild-type; +: integrated defense system; Δ: knockout of the defense system; cl: clone. Source data are provided as a Source Data file.
