# Supplementary material for: Strengthening phage resistance of Streptococcus thermophilus by leveraging complementary defense systems
Source: Nat Commun. 2025 Aug 4;16:7142. doi: 10.1038/s41467-025-62408-3 (PMC12322248; doi:10.1038/s41467-025-62408-3)
Supplement: Supplementary file 2 — Description of Additional Supplementary Files [file 41467_2025_62408_MOESM2_ESM.docx]

**Description of Additional Supplementary Data**

**Title: Supp. Data 1**

**Description: *S. thermophilus* genomes used in the bioinformatic analysis.** Genomes were downloaded from Refseq in March 2023. Plasmids were identified with Abricate while prophages were detected with Phigaro and confirmed with Phastest.

**Title: Supp. Data 2**

**Description: Defense systems identified in *S. thermophilus* using PADLOC and DefenseFinder.** Raw data from the bioinformatic analysis excluding candidate systems and those annotated as “other” and “cas_adaptation”. The accession number of each protein in each defense system is listed in the “Protein in the defense system”. Proteins that do not begin with “WP” indicate that the related defense system gene is a pseudogene.

**Title: Supp. Data 3**

**Description: Crosstab table of defense system counts in *S. thermophilus*.** This table lists the number of each defense system present in each genome. In the column "CRISPR-II-A" a value of 1 indicates the presence of a CR1 locus only, while a value of 2 indicates the presence of both CR1 and CR3 loci. This crosstab corresponds to the heatmap shown in Fig. S1.

**Title: Supp. Data 4**

**Description: Detailed information on phages used in the bioinformatic analysis.** Genomes were downloaded from NCBI in June 2023. This table includes data on their genus, GC content, genome length, and the presence of methylase and anti-CRISPR (ACR) proteins.

**Title: Supp. Data 5**

**Description: Prevalence of defense systems found in *S. thermophilus* across other species, genera, and phyla, based on DefenseFinder results.** The numbers were retrieved from DefenseFinder RefSeq database (<https://defensefinder.mdmlab.fr/wiki/refseq>), which compiles DefenseFinder results for the bacterial RefSeq database of complete genome as of May 2022 (accessed in December 2024). In this table, defense systems are categorized according to DefenseFinder, which leads to differences compared to Fig. 1A, where results also include PADLOC predictions and data from incomplete genomes. Notably, Abi2 and AbiD, which predominantly match the same genes, were clustered as AbiD systems in our analysis and the closely related PD-T7-2 and Gao19 were merged into a single system denoted as Gao19. † and ‡ indicate defense systems identified only by PADLOC or found exclusively in incomplete genomes respectively, with their counts (in **bold**) reflecting our own analysis.

**Title: Supp. Data 6**

**Description: InterPro functional annotation of the defense systems found in *S. thermophilus*.**

**Title: Supp. Data 7**

**Description: Statistical analysis of the phage resistance efficacy when defense systems were combined.** The synergy score was calculated from the Area Under the Curve (AUC) obtained for each tested condition during the liquid culture assays. This score represents the difference between the AUC of the combination (e.g., CR1[Gabija]) and the sum of the AUC for each defense system (CR1 + Gabija). The statistical significance (p-value < 0.05) of the synergy scores was evaluated from three biological replicates using a two-sided one-sample t-test with Benjamini-Hochberg correction for multiple testing. A positive score indicates a synergistic effect, while a zero score reflects an additive effect of the defenses systems. A negative score indicates that the combined systems do not provide enhanced resistance compared to either system used alone or that there is antagonistic effect.

**Title: Supp. Data 8**

**Description: Detailed information on identified restriction-modification (RM) systems in *S. thermophilus*.** Recognition sites were predicted with REBASE. “N.D” indicates that the protein was not used for the analysis, while "None" specifies the absence of the corresponding subunit in the RM system. The "Count" column represents the number of times each homolog is found in *S. thermophilus* genomes.

**Title: Supp. Data 9**

**Description: Strains and plasmids used in this study.**

**Title: Supp. Data 10**

**Description: Primers used in this study.**
